# Active Learning and Professional Citizenship in Nursing Education: A Structural Account

**DOI:** 10.1111/nup.70113

**Published:** 2026-08-31

**Authors:** Maria Efstathiou, Mary Gouva, Elena Dragioti

**Affiliations:** ^1^ Scientific Laboratory of Psychology and Person‐Centred Care, Department of Nursing, School of Health Sciences University of Ioannina Ioannina Epirus Greece

**Keywords:** active learning, deliberative pedagogy, nursing education, professional citizenship, professional formation

## Abstract

Active learning and professional citizenship are widely discussed in nursing education, yet the conditions under which they converge have not been theoretically specified. In this paper, we advance a structural account of that relation, drawing on deliberative theory, philosophy of education, and nursing scholarship. The relation we describe is one of shared enabling conditions rather than causal production: active learning and professional citizenship converge not because one produces the other, but because each depends on arrangements that require public justification through reasons, engagement with competing claims, and answerability for the consequences of action. We trace a conceptual movement from professional accountability to professional citizenship, and from participatory learning to deliberative practice, specifying a set of conditions that can be instantiated in nursing education but have rarely been theorised as the basis of this relation. In doing so, we give theoretical form to a relation more often enacted than articulated.

## 1 Introduction

Education is not only the passing on of established knowledge but the formation of the capacities through which people think, judge, and take part in common life (Russell 1926/2023). What a generation hands on is not a fixed possession but a living inheritance, reshaped by those who take it up (Fontal and Gómez‐Redondo 2016). Professional education, and nursing in particular, raises the question of whether and how learning cultivates not only competence but also orientations toward belonging, accountability, and participation in a shared social world. The question pursued here is not what nursing education should include, but whether the terms on which learning is organised allow such orientations to arise.

This question arises within a contemporary educational setting marked by two related developments. Competency‐ and outcome‐based frameworks, which state what graduates must be able to do in observable and assessable terms, have acquired a prominent place in nursing curricular design (Mani 2025; Mueller‐Burke et al. 2024), while active, collaborative, and practice‐oriented approaches are promoted as ways of preparing students for the complexity of clinical work (Bingen et al. 2023; Waltz et al. 2014). These developments answer legitimate demands for quality and accountability, making the preparation of nurses legible to regulators, employers, and students. They do not, however, by themselves specify the terms under which students learn to exercise judgement among peers, patients, and colleagues, whose claims they must weigh and to whom they become answerable. It is within this unresolved dimension that the present enquiry is situated.

Professional formation in nursing involves more than acquiring disciplinary knowledge. It requires practical wisdom, or phronesis in the Aristotelian tradition, understood as the capacity to deliberate about what a particular situation requires and to act well, through judgement that cannot be reduced to the application of general rules. Jenkins et al. (2019) take up this conception within nursing, arguing for the primacy of professional deliberation and judgement in good practice and treating phronesis as a central dimension of nursing education.

This deliberation rests on a form of clinical knowledge that is interpretive. Tanner (2006) describes clinical judgement as a process shaped by what the nurse brings to the encounter: prior experience, knowledge of the individual patient, and the context of care. Analytic reasoning is not abandoned but situated within the wider activity of noticing, interpreting, responding, and reflecting. Kinsella (2010) extends this account by treating reflective practice as professional knowledge generated in action rather than received in advance of it.

Expertise develops over time. Benner (1984) presents its formation as emergent rather than linear, built through immersion in practice and the gradual internalisation of responses refined across repeated clinical encounters. This account concerns the developmental pathway through which clinical expertise emerges rather than the epistemological character of the knowledge involved.

Three claims emerge from this discussion: nursing calls for deliberation about what a particular situation requires; the knowledge on which such deliberation rests is interpretive; and the capacity to exercise it develops through sustained engagement with practice. These claims are, respectively, normative, epistemological, and developmental. Together, these accounts point to a distinctive understanding of learning. A capacity that consists of judgement cannot be cultivated where learners have no opportunity to exercise judgement. Here, active learning refers broadly to educational arrangements in which learners participate in interpreting and using knowledge rather than receiving it only (Prince 2004); whether such participation becomes deliberative is the further question pursued below. Active learning is therefore not simply one teaching approach among many; it corresponds to a form of professional practice in which knowledge, judgement, and experience come together in responding to clinical situations.

The literature on active learning and professional citizenship has largely developed apart. Active learning has been studied chiefly in relation to pedagogical outcomes and, within the traditions of service and civic education, linked to public engagement and active citizenship (Harris 2010; Hollister 2006). Professional citizenship, by contrast, has been treated as a standing, a disposition, or a form of engagement belonging to the individual practitioner (Fulton 2020; Huston 2025). The present account reorients this notion toward the relational and answerable dimension of professional practice, a sense developed below in relation to accountability.

The argument developed here departs from both lines of work: the study of active learning in terms of pedagogical outcomes, and the treatment of professional citizenship as an individual standing or disposition. Its aim is not to show that active learning produces professional citizenship, nor that the two arise from a common antecedent. By a structural relation, we mean one that holds at the level of enabling conditions rather than causal effects: active learning and professional citizenship converge because each depends on arrangements that require public justification through reasons, engagement with competing claims, and answerability for the consequences of action. These conditions are not stipulated as definitional features of either concept; their philosophical basis is developed below through Arendt's account of action.

The question, then, is not whether active learning leads to professional citizenship, but under what educational conditions professional action acquires a civic character. This is a conceptual question rather than an empirical one: it asks not whether participatory learning improves educational outcomes, but what such a character presupposes. Nursing practice unfolds within institutional systems, yet its object is not the delivery of care alone but the person situated within a wider social world. This relational character has long been recognised in nursing scholarship, where clinical reasoning is treated as a defining feature of practice and professional accountability as a central commitment of safe and ethical care (Krautscheid 2014; Oyetunde and Brown 2012). Answering the question, therefore, requires moving beyond educational outcomes to the conditions under which professional action assumes a deliberative and civic form.

If professional formation takes shape in relation to others, education cannot remain indifferent to the settings in which deliberative capacity develops. Drawing on Arendt, Nixon (2020) argues that thinking, judgment, and action are formed within a common world rather than in isolation, making education answerable for the conditions under which these capacities emerge. From this perspective, active learning provides situations in which learners must exercise judgement, respond to peers, and justify their decisions before responsibility for practice becomes their own. Lind (2015) reinforces this educational argument by showing that moral judgment competence may regress when opportunities for responsibility‐taking and guided reflection are systematically absent.

Professional formation, understood in these terms, involves a shift in how practitioners understand their own action: from an ‘I‘‐centred perspective toward the recognition that action always takes place among others, without dissolving individual agency. Professional identity is not displaced by this movement. It takes shape within the relational settings in which care inevitably unfolds. In nursing, this relational character is not incidental but intrinsic. Clinical action unfolds between the nurse, those in care, colleagues, and the institution. The civic dimension is therefore not added to practice from outside; it is already implicit in these relations.

What follows for professional education concerns the form of learning rather than its content. Where learning is organised through participation under deliberative conditions, students are no longer positioned simply as recipients of knowledge but as actors who assume responsibility within a common professional life. On this account, active learning and professional citizenship are grounded not in shared outcomes or competencies but in the conditions under which practical wisdom, participation, and civic responsibility become possible. Where these conditions are absent, learning and professional action may still occur, but neither takes on a deliberative or civic form. On this basis, we develop a conceptual account of professional citizenship in relation to accountability and examine how participatory forms of learning render its formation visible within nursing education.

## 2 Transmission and Participation: Repositioning Professional Formation in Nursing Education

Transmission and participation are treated here as analytically distinct orientations rather than mutually exclusive models of professional formation. In transmission‐oriented arrangements, learning follows a vertical logic: knowledge is organised, presented, and assimilated within clearly differentiated instructional roles, an arrangement Freire (2018) described as the “banking” conception of education, in which learners are treated as receptacles rather than agents. Such arrangements can support clarity, disciplinary coherence, and the acquisition of established knowledge and skill. Yet they organise formation as a largely monological process, in which knowledge moves from instructor to learner and the learner's role remains largely receptive (Reznitskaya and Gregory 2013). Their limitation for the present argument lies elsewhere: when learning is organised primarily as a one‐directional flow, learners have limited opportunity to exercise judgement, expose it to scrutiny, and justify it. Writing on digital capability in nursing, Wynn (2025) treats this dimension of formation as a complement to technical competence rather than a substitute for it.

Participation reconfigures learning as a shared process in which understanding develops through interaction rather than reception alone. A paradigmatic account of this orientation is Freire's problem‐posing education. The present argument draws specifically on its pedagogical logic without adopting the broader emancipatory project in full: learners interrogate knowledge rather than simply receiving it, and the questions they raise become part of the material of inquiry rather than interruptions to it (Freire 2018). Teacher and learner occupy distinct positions, yet understanding develops through their interaction rather than being simply passed from one to the other, so that each contributes to what comes to be known (Leclerc‐Loiselle et al. 2019). Understanding adequate to practice can be cultivated in this way, through engagement rather than delivery, within the recursive movement Freire called praxis, in which reflection and action continually inform one another.

In nursing, this way of organising learning takes a particular form because professional practice draws on modes of knowing that resist reduction to technical application or rule‐following. Carper (1978) account of empirical, aesthetic, personal, and ethical patterns of knowing describes a discipline structured by distinct and complementary modes of engagement with practice. Johns (1995) extended this account by showing how structured reflection can bring multiple patterns of knowing into dialogue within practice. A predominantly monological model offers limited scope for cultivating this multiplicity. These patterns are not separable bodies of content to be delivered in sequence; they are cultivated as learners interpret experience, encounter different perspectives, and test their judgments in dialogue with peers, educators, and those receiving care.

Participation alone does not make learning deliberative or civic. Practical reasoning acquires that character when learners are required not only to contribute but to justify their judgements, weigh competing claims, and remain answerable for the consequences that follow. This civic character does not arise because civic content has been added to the curriculum, but because such educational situations instantiate demands that also structure professional action. The discussion now examines this correspondence through the distinction between professional accountability and professional citizenship.

## 3 Professional Accountability and Professional Citizenship: A Conceptual Clarification

Professional accountability occupies a central place in nursing discourse, shaping how professional integrity and reliability are understood. Although treated as foundational, accountability has been described in the literature without a consistent definition, a difficulty that has complicated its teaching and evaluation within nursing education (Krautscheid 2014). Accountability designates the point at which professional action becomes answerable to publicly recognised standards of practice and where individual judgement enters a shared evaluative framework. In nursing, accountability has been conceptualised as a three‐dimensional value: taking responsibility for one's decisions and actions, making them transparent, and agreeing to be judged in accordance with society's accepted values (Drach‐Zahavy et al. 2018). It is not incidental to professional formation; it is one of its primary aims.

Yet answerability to established norms, however necessary, does not fully account for the moral demands of professional life. Nursing practice is relational not only in the nurse‐patient relationship but also in the work nurses do in facilitating and coordinating care within complex organisational networks. Liaschenko and Peter (2004) draw an epistemological consequence from this: the social organisation of care shapes which concerns become visible as moral and whose standing it is to name them, so that an ethics centred on professional accountability constrains the claims that register as moral. The responsibility at stake, however, is not created by institutional standards. It is formalised through accountability but not exhausted by it. Professional action can meet its institutional standards while remaining indifferent to the structural conditions that return the same patients, repeatedly, to the same ward. Such action is accountable in the terms the framework recognises, yet those conditions are not among the things accountability ordinarily requires practitioners to answer for. And such action does not thereby assume the fuller civic dimensions of practice.

Professional citizenship has been articulated in nursing partly through the language of membership. Fulton (2019) traces the concept to citizenship in the ancient polis, where individual standing was inseparable from participation in the life of the community, and presents professional citizenship as membership in a community that carries rights of participation together with corresponding responsibilities. On this account, belonging provides the standing from which such engagement becomes both possible and expected. Huston (2025) places greater emphasis on enactment, locating professional citizenship in what practitioners actively do, both within nursing and in the wider community, to confront problems that persist over time. This shift matters because membership does not by itself ensure participation; Huston notes a decline in such engagement among those who nevertheless retain professional standing. Although they differ in emphasis, both accounts locate the individual practitioner as the primary bearer of professional citizenship, whether through membership or conduct. The present account shifts the unit of analysis. Professional citizenship is not a property of the practitioner taken alone; it is constituted where practice requires public justification, engagement with competing claims, and answerability for the consequences of action, and it is therefore inseparable from the educational and institutional arrangements under which professionals learn and work. The civic character at issue here is not, in the first instance, political advocacy or engagement beyond the clinic. It is the justificatory and relational structure internal to ordinary clinical judgement: the requirement to give reasons, to engage competing claims, and to remain answerable for the consequences that follow. Advocacy and public engagement may express this orientation, but they do not constitute it.

This is where the distinction between accountability and professional citizenship becomes consequential. Where accountability asks whether existing standards have been met, professional citizenship asks what those standards should be and what responsibility practitioners bear for shaping them, a shift Huston (2025) describes as a move from policy implementation to policy development. The former directs professional action toward congruence with established standards (Drach‐Zahavy et al. 2018); the latter directs it outward, toward the social and political barriers that limit what people are able to be and to do (Lazenby 2020). In nursing, this outward orientation is not an optional extension of professional life but an expression of what clinical action already is: the coordination of care across relational and organisational networks already has a public dimension, because it brings distinct actors, needs, and claims into a field of shared responsibility. The two are not opposed: accountability is a necessary condition of professional citizenship, but it is not a sufficient one.

Professional citizenship takes dispositional form in practitioners, yet it is not a discrete competency, nor is it cultivated through instruction and curricular content alone; rather, it becomes established as a habit of mind through sustained experience (Fulton 2020), in situations that demand ethical reasoning, perspective‐taking, and collective deliberation. Accountability frameworks, which assess whether standards have been met, do not by themselves constitute such situations. Professional citizenship becomes possible under educational conditions in which learners are required to exercise judgement with and before others.

Recent work in nursing education points in this direction. Chiangkhong et al. (2026) examine community health practicums as sites that place students in emotionally and ethically demanding dilemmas, where reflective practice develops what the authors describe as relational understanding, an attentiveness to the social and systemic dimensions of care. Similarly, Einhellig et al. (2015) found that an affective learning strategy, a poverty simulation, shifted students' explanations of poverty away from personal attributes and toward structural and social determinants, a movement the authors link to social justice as a professional nursing value. Taken together, these studies illustrate pedagogical contexts in which learners encounter genuine uncertainty, reason in the presence of perspectives and claims beyond their own, and reconsider their judgements against a wider frame than the individual case alone. Read through the structural account developed here; these are contexts in which active learning may acquire a deliberative character.

## 4 Active Learning as Deliberative Practice

Active learning is frequently associated with student engagement, active participation, and instructional variety. Prince (2004) defines active learning broadly as instructional methods that engage students in the learning process through meaningful activity and reflection on what they are doing. Such activity does not by itself determine the deliberative character of the learning. Students may exchange ideas, collaborate, and contribute to classroom tasks without being required to justify their judgments publicly, to engage with competing claims, or to reconsider their positions in light of better reasons. Active learning becomes deliberative practice only when participation is organised under conditions that render judgment answerable rather than merely expressed (Englund 2006). Englund himself rejects the view that deliberative communication stands opposed to the transmission of knowledge, holding that it can contribute to knowledge‐formation in its own right, and he connects it directly to active learning, since the confrontation of differing views can sharpen a participant's reasoning and lead to a change of standpoint.

In the account developed here, that structure of answerability rests on three jointly constitutive conditions. Positions must be publicly justified in the presence of those who may contest them, and competing claims must be engaged rather than evaded or dismissed (Englund 2006; Gutmann and Thompson 2000). A third condition concerns what follows from judgement: judgements must remain answerable for the consequences to which they give rise. To justify a position is already to acknowledge that contestation is possible; to remain answerable for what follows from a judgement is to accept that its adequacy must also be tested against the foreseeable consequences of acting on it. What this structure produces is not necessarily agreement but a form of exposure: reasoning becomes visible, and its adequacy becomes a matter that those present may legitimately assess. Deliberation on this account requires that judgment remain open to revision when its justification proves insufficient (Gutmann and Thompson 2000). In the pedagogical setting, consequential answerability is rehearsed through revisability: learners can be required to trace the consequences that follow from a judgement and, where these expose inadequacies in its justification, to revise it. Englund (2006) treats deliberative communication and deliberative democracy as differing in degree rather than in kind, and characterises the classroom as a setting marked by asymmetries of knowledge, experience, and authority. The two registers, on the account given here, realise a single structure of answerability under different conditions.

This understanding of deliberation is not confined to civic education. Cognate formulations appear in medical education, where Barilan and Brusa (2013) argue that neither formal codes nor virtue‐oriented professionalism is adequate in isolation, and present moral deliberation as an overarching competency that accompanies and integrates the development of more specific professional competencies. On their account, deliberation does not stand apart from knowledge and standards. Participants examine their considered judgements in the light of ethical theory and codes of practice, test both against the case and its narrative, and remain open to differing voices and sensibilities. Deliberation is therefore not content‐free: it draws on relevant facts, bodies of knowledge, professional standards, and competencies, while subjecting their relevance and application to critical examination.

The resources on which it draws are not of a single kind. Codes and standards supply normative constraints and reasons; competencies designate the capacities through which knowledge and standards are interpreted and enacted. Deliberation concerns how these resources are brought to bear in situations in which their application itself requires judgment. It is not, on this account, coextensive with clinical reasoning. Clinical judgement also depends on substantive knowledge, interpretation of the particular situation, and technical competence; deliberation becomes salient where evidence, standards, or competing values leave more than one defensible course of action open. Codes and competencies are thus not rivals to deliberation but among the resources on which deliberative judgement depends.

Writing about professional education more broadly, Trede and McEwen (2016) describe deliberate professionals as attentive to the relationship between competent skill mastery and the capacity for deliberation and deliberate action. They also caution that participation and dialogue do not by themselves secure deliberative learning, since these can reproduce established practices when the assumptions and value frameworks that sustain them go unquestioned. Taken together, these accounts indicate that professional formation is not exhausted by the acquisition of competencies, and that deliberation does not displace them. It concerns the conditions under which competent action becomes reflectively judged and answerable.

Related strands of nursing scholarship bear on this terrain without occupying it. Cestari (2002) draws on Habermas's theory of communicative action to argue for a dialogical form of teaching and learning in nursing education, while Gehrke (2008) addresses the preparation of nurses for civic engagement, understood as participation in the political and policy processes through which health is shaped. These lines of work do not themselves constitute a unified theory of deliberative learning. The contribution advanced here is narrower: to specify the conditions under which participation becomes deliberative, such that judgment must be justified, exposed to relevant disagreement, and held answerable for what follows from it. Active learning, on this account, is deliberative not because it increases participation, but because educational arrangements can place competent judgement under these demands. Once those conditions are made explicit, a further question arises: whether they belong to the organisation of learning alone, or also to the form of professional action for which nursing education prepares.

## 5 From Educational Configuration to Civic Deliberation

In nursing, deliberation is not confined to pedagogical arrangements but takes shape within practice itself, where reasoning is not only articulated but carries weight. Discussion may accommodate differences; deliberation, however, organises engagement with them. Participants are called to justify what they propose by offering reasons that those bound by the resulting decision could reasonably accept (Gutmann and Thompson 2000). Englund (2006) extends this account into educational contexts: deliberative communication requires participants to take a stand while listening to, seeking, and evaluating arguments, and to strive collectively toward values and norms on which those involved can agree. In nursing education, Goodin and Stein (2008) give this structure a concrete pedagogical form. They describe deliberative discussion as a shared inquiry in which participants talk through and weigh the costs and consequences of alternative responses to a public problem. Shared norms, so understood, are not a precondition of deliberation but its provisional achievement: agreements remain temporary, and deliberation may equally end in the recognition that agreement cannot be reached.

These conditions acquire pedagogical form when learning is deliberately organised around them. In professional education, this organisation cannot be left to chance: preparing students for professional responsibility requires spaces, established early in professional programmes, in which they develop the ability to deliberate on different alternatives, try them out in practice, and reflect upon them (Solbrekke and Englund 2011). Active learning, understood as deliberative practice in the sense developed above, does more than encourage participation; it reorganises the terms under which participation becomes consequential. Its deliberative character depends on whether disagreement becomes visible and the articulation of reasons becomes unavoidable. In a case study of a doctoral higher education policy course, students described public deliberation as enabling them to encounter multiple perspectives and weigh the trade‐offs associated with alternative courses of action (Johnson et al. 2014). Within such an arrangement, the learner becomes answerable not only for the adequacy of a judgement but also for the consequences that follow from it within a shared world. Without these conditions, professional formation may still support competent performance; that achievement alone, however, does not make civic orientation a structural feature of practice.

In Arendt's terms, much of what nursing involves belongs to labour and work: bodily care that answers to the necessities of life and technical procedures directed toward determinate ends (Arendt 1958/1998). Both are indispensable. Arendt's distinction has already been brought into nursing by Sousa et al. (2020), who use labour, work, and action to interpret the human and occupational condition of nurses in Psychosocial Care Centres and locate action in the intersubjective relations among nurses, institutions, and service users. The present account takes this nursing‐specific application in a different direction: rather than treating the triad primarily as a description of the nurse's occupational condition, it develops the space of appearance, plurality, and irreversibility as the Arendtian terms corresponding to the three conditions identified here (Arendt 1958/1998). The claim is not that nursing is a pure form of action, but that within it the structure of action cannot be evaded. This is not simply because care unfolds among people, but because clinical judgement enters a field in which it must be disclosed before others, exposed to competing claims, and taken up in decisions whose consequences cannot always be fully undone. Those affected by clinical decisions, and those who share responsibility for care, can therefore ask for, hear, and weigh the reasons offered for them. Where a decision bears on someone who suffers, the demand to give reasons is not merely an external formality but internal to the encounter. Plurality, in turn, is not abstract: it appears in the patient whose account of pain resists clinical categorisation, in the family whose understanding diverges from the treatment plan, and in the interprofessional team, where professional roles and the balance of power remain ethically contested (Pakkanen et al. 2022). To act among these accounts is to engage the claims they raise rather than to set them aside. Consequently, finally, it is not metaphorical: what is decided at the bedside cannot always be undone, so that the one who acts remains answerable for what follows from it. These are the three conditions with which we began. Public justification, engagement with competing claims, and answerability for consequences correspond, respectively, to the space of appearance, plurality, and the irreversibility of action. This correspondence locates the convergence of deliberative learning and professional citizenship at the level of structure rather than causal production or shared outcomes. Nursing is, therefore, a field in which the structure of action cannot be treated as incidental.

This structure can nevertheless be obscured when care is framed primarily as the production of measurable outcomes. Kohlen (2015) shows how care practices may be fragmented into ‘countable pieces of workloads,’ leaving less space for action as a praxis of the unpredictable. In Arendt's terms, this is a displacement of action by work. Thomas et al. (2020) identify a related tension between the demand to meet performance indicators and attending to patients as unique individuals. Under such productivity pressures, what can be recorded may acquire greater institutional visibility, and care becomes more readily visible as output than as engagement. This reframing is not without function. Measurement can offer safety, comparability, and a form of accountability toward those outside the immediate encounter; these are real goods, and no account of care can dispense with them. Its appeal is structural: what can be counted can be compared, audited, and defended, whereas deliberative judgement cannot be fully rendered in a metric of the same kind. The difficulty is not that care is measured, but that care is not exhausted by what can be measured, and the problem begins where the measurable becomes the only register in which practice is visible. The same asymmetry can shape how education is organised: outcomes that can be specified and compared are institutionally more legible than forms of judgement that resist such specification. A reasoning‐centred formation may therefore be disadvantaged even where its value is acknowledged. On this account, deliberative spaces may be displaced not because their value is denied, but because they require time, interpretive attention, and forms of shared judgement that are difficult to standardise and remain less institutionally legible within productivity and audit systems.

The same displacement of action by work reaches into the institution itself, narrowing the occasions on which reasoning about care can be raised at all. Moral distress has classically been described as the situation in which a nurse judges what the ethically right course of action would be while institutional constraints prevent the nurse from acting on it (Jameton 1984). Read structurally, this constraint is not simply an obstacle standing between judgment and action; it is the absence of an effective institutional point at which judgment can become public and bear on what is done. Ramos et al. (2020) sharpen this reading by describing moral distress as an obstruction of the deliberative process that leaves it inconclusive. Moral distress arises, then, not only from ethical complexity but from the inability to move concerns into forums where they can be articulated and carry weight. This inability is not primarily psychological but structural. Where institutional arrangements offer no effective space for reasoning to become publicly visible and answerable, moral distress is not a matter of individual inadequacy but of the absence of the very conditions that deliberative action requires. In Arendtian terms, this is the deprivation of a shared public realm within the institution itself.

The question of what an institution organised otherwise would look like, therefore completes, rather than departs from, the argument. This is not to deny that institutions provide occasions for ethical reflection. Ethics committees, interprofessional rounds, and reflective‐practice programmes offer recognised settings in which concerns can be raised. The limitation is structural rather than a matter of absence: such settings tend to be specialised, convened episodically, and set apart from the point at which judgement must bear on what is done, so that reasoning about care remains confined to designated forums rather than able to enter its ordinary conduct. Liaschenko and Peter (2016), drawing on Walker's formulation, argue that health care institutions can themselves be moral communities: places, both literal and figurative, that keep ‘moral space open.’ In such institutions, moral language can flourish in ordinary conversations, meetings, classes, and rounds rather than being confined to ethics committees, and nurses can claim the standing to have their concerns taken seriously because their contribution to care matters. An institution of this kind satisfies precisely the conditions under which, as the preceding sections have argued, deliberative learning and professional citizenship are each constituted: judgement becomes visible, competing claims are engaged, and action remains answerable for its consequences. Empirical work in primary health care points in the same direction. Nora et al. (2015) found that ethical problems subject nurses to moral suffering, that nurses report lacking structural support to discuss and resolve them, and that structured deliberation is recognised by nurses themselves as a way of coping. These findings do not establish that deliberative forums remove moral distress. They show, however, that nurses identify the absence of structural support for ethical discussion as part of the problem and structured deliberation as one possible response.

It is at this point that the pedagogical significance of the argument becomes clear. A learning arrangement organised as deliberation does not import civic content into nursing education from outside; it makes explicit the relational and justificatory demands already present in nursing action, the same demands with which this section began. Recent curricular work on the formation of ‘citizen nurses’ shows how this orientation can be given concrete educational form. Clark et al. (2023) describe an experiential curriculum in which students apply civic studies and develop their capacity to co‐create change with the communities they serve, and a subsequent qualitative evaluation found that students described a changed understanding of what it means to be a nurse and reported feeling better prepared to take action (Clark et al. 2024). These findings do not establish that such preparation transfers into professional practice, but they show that students experienced the curriculum as preparation for civic action. Holmes and Warelow (2000) offer a complementary normative warrant: conceiving nursing as praxis that is profoundly political, and ethical praxis as a form in which clinical expertise embodies ethical principles rather than having them appended from without. Professional citizenship, on this account, is neither exhausted by an identity to be assumed nor reducible to an outcome to be assessed; it is the orientation that a deliberative formation renders visible and gives students practice in sustaining.

## 6 Conclusion

What is at issue is not the addition of a new pedagogical aim, but the recognition that civic orientation cannot become a structural feature of professional formation where the conditions of deliberation are absent. The question is therefore not what nursing education should include, but whether the terms on which learning is organised allow it to take shape.

This structural relation concerns not only curriculum design but the terms under which practitioners learn to act. Where disagreement is exposed, and reasons must be given publicly, competence is formed within an orientation toward a shared world rather than apart from it. Professional citizenship, in this sense, is constituted by the very conditions that constitute active learning as deliberative practice.

## Author Contributions

**Maria Efstathiou:** conceptualisation, investigation, writing – original draft. **Mary Gouva:** critical review and editing. **Elena Dragioti:** supervision, investigation, theoretical review, conceptualisation.

## Funding

The authors have nothing to report.

## Ethics Statement

Not applicable. This paper is a philosophical essay and does not involve human participants, personal data, or interventions requiring ethical approval.

## Conflicts of Interest

The authors declare that they have no known competing financial interests or personal relationships that could have appeared to influence the work reported in this paper.

## Data Availability

Data sharing not applicable to this article as no datasets were generated or analysed during the current study.
